# Thoracoscopic-assisted repair of a bochdalek hernia in an adult: a case report

**DOI:** 10.1186/1752-1947-4-366

**Published:** 2010-11-17

**Authors:** Noriaki Tokumoto, Kazuaki Tanabe, Hideki Yamamoto, Takahisa Suzuki, Yoshihiro Miyata, Hideki Ohdan

**Affiliations:** 1 Department of Surgery, Division of Frontier Medical Science, Programs for Biomedical Research, Graduate School of Biomedical Sciences, Hiroshima University, 1-2-3 Kasumi, Minami-ku, Hiroshima, 734-8551; 2 Department of Surgical Oncology, Research Institute for Radiation Biology and Medicine, Hiroshima University, 1-2-3 Kasumi, Minami-ku, Hiroshima, 734-8551, Japan

## Abstract

**Introduction:**

Bochdalek hernia is a congenital defect of the diaphragm that usually presents in the neonatal period with life-threatening cardiorespiratory distress. It is rare for Bochdalek hernias to remain silent until adulthood. Once a Bochdalek hernia has been diagnosed, surgical treatment is necessary to avoid complications such as perforation and necrosis.

**Case presentation:**

We present a 17-year-old Japanese boy with left-upper-quadrant pain for two months. Chest radiography showed an elevated left hemidiaphragm. Computed tomography revealed a congenital diaphragmatic hernia. The spleen and left colon had been displaced into the left thoracic cavity through a left posterior diaphragmatic defect. We diagnosed a Bochdalek hernia. Surgical treatment was performed via a thoracoscopic approach. The boy was placed in the reverse Trendelenburg position and intrathoracic pressure was increased by CO_2 _gas insufflations. This is a very useful procedure for reducing herniated contents and we were able to place the herniated organs safely back in the peritoneal cavity. The diaphragmatic defect was too large to close with thoracoscopic surgery alone. Small incision thoracotomy was required and primary closure was performed. His postoperative course was uneventful and there has been no recurrence of the diaphragmatic hernia to date.

**Conclusion:**

Thoracoscopic surgery, performed with the boy in the reverse Trendelenburg position and using CO_2 _gas insufflations in the thoracic cavity, was shown to be useful for Bochdalek hernia repair.

## Introduction

Congenital diaphragmatic hernias (CDHs) occur when muscular portions of the diaphragm fail to develop normally, resulting in the displacement of abdominal components into the thoracic cavity [1]. CDHs occur mainly during the eighth to the tenth weeks of fetal life. They consist of Bochdalek, hiatal and Morgagni hernias. Bochdalek hernias, caused by posterolateral defects of the diaphragm, were first described by Bochdalek in 1848 [2]. They usually present with severe respiratory distress immediately after birth, which is life-threatening. Once diagnosed, Bochdalek hernias should be surgically treated during the neonatal period. Therefore, adult cases are rare, with a reported frequency of 0.17% to 6% among all diaphragmatic hernias [3,4].

We performed minimally invasive surgery under thoracoscopic guidance, for an incidentally diagnosed Bochdalek hernia in an adult [5,6]. We describe the surgical procedures for thoracoscopic-assisted Bochdalek hernia repair and its advantages and disadvantages.

## Case presentation

**Figure 1:**
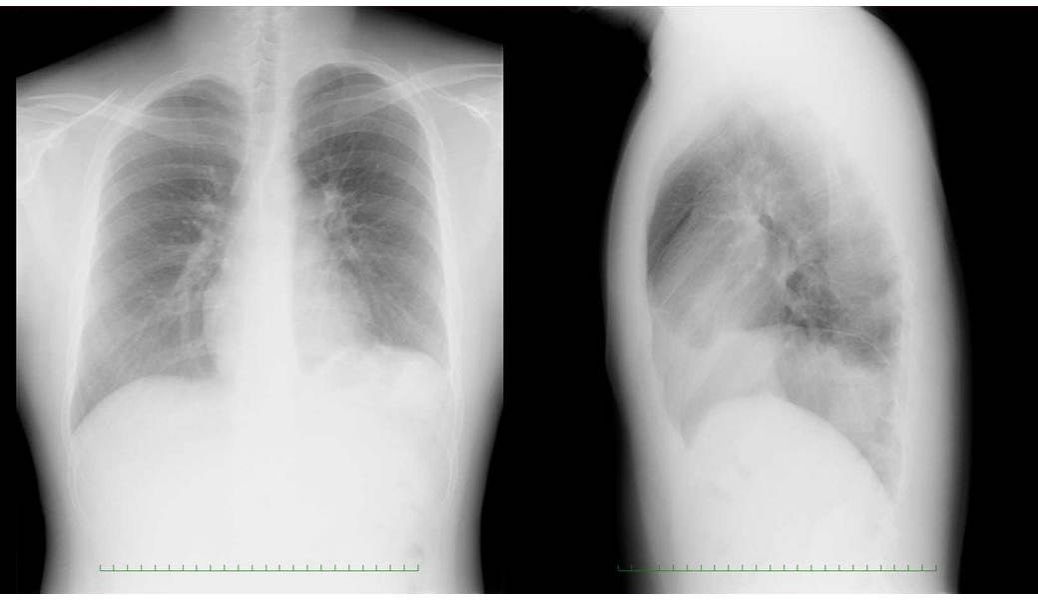
**Preoperative chest radiograph**. The chest radiograph shows elevation of the left diaphragm. In this case, the lateral chest radiography was important for the detection of an abnormality in the thoracic cavity.

**Figure 2:**
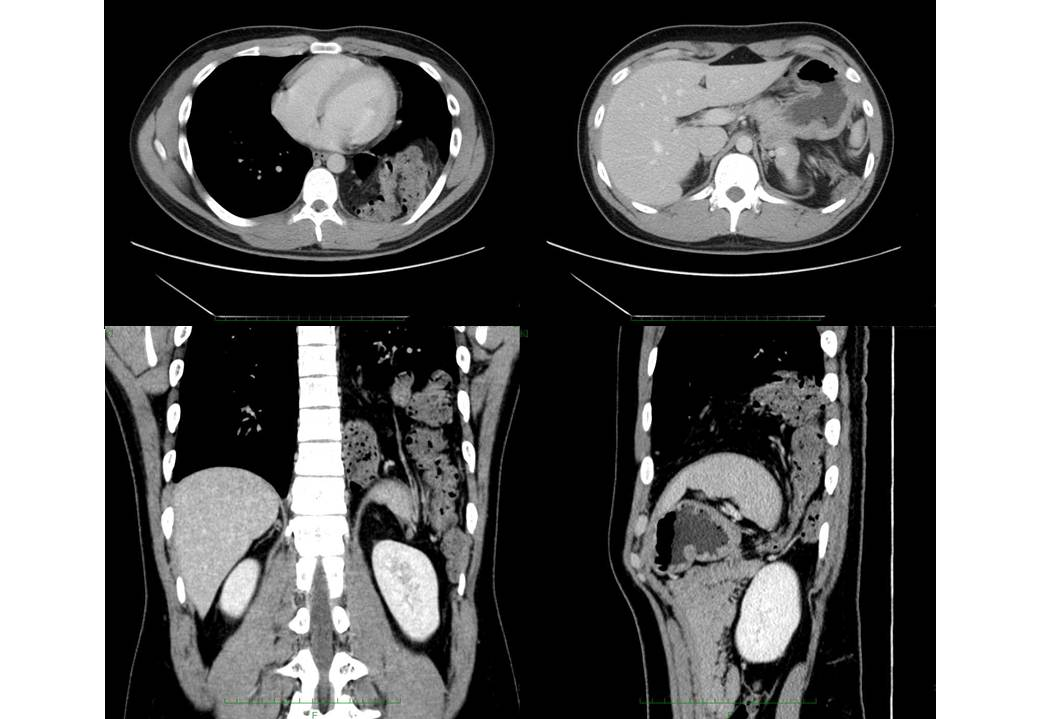
**Preoperative enhanced chest and abdominal computed tomography (CT) scans**. The chest CT shows a diaphragmatic hernia. The spleen and left colon have herniated into the left thoracic space through a left posterior diaphragmatic defect.

A 17-year-old Japanese boy was referred to our hospital with a suspected CDH. He had experienced occasional left-upper-quadrant pain for two months. The pain then intensified and occurred more often. He consulted a neighborhood clinic, and was referred to our hospital. There was no history of trauma. Chest radiography showed elevation of the left diaphragm (Figure 1). Computed tomography (CT) of the chest revealed CDH (Figure 2). The spleen and left colon had herniated into the left thoracic space through a left posterior diaphragmatic defect. We therefore diagnosed the patient as having a Bochdalek hernia.

**Figure 3:**
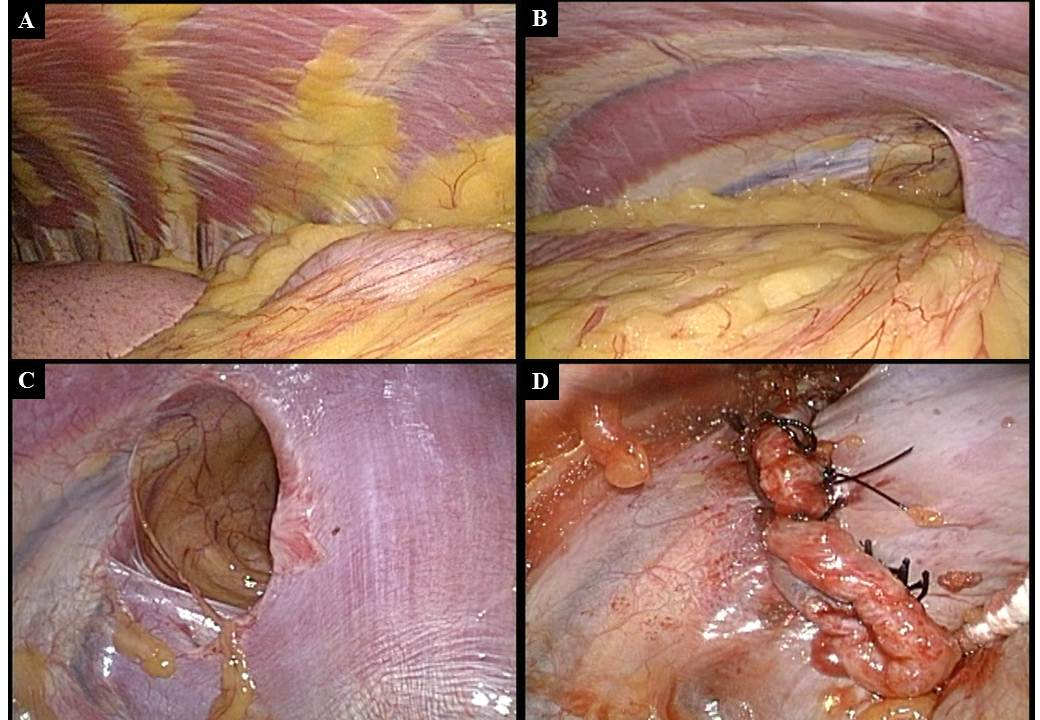
**Surgical findings with thoracoscopy**. A) The left colon and spleen were identified in the left thoracic cavity under thoracoscopy. No hernia sac was found. B) The left colon and spleen appeared to have herniated through a left posterior diaphragmatic defect, as indicated by the preoperative chest computed tomography. C) The diaphragmatic defect, 5 cm × 6 cm in size, had a smooth circular edge and showed gradual expansion at the thoracic wall. D) The defect was closed using a single layer primary closure method with interrupted non-absorbable sutures.

He was prepared for surgery via a left thoracoscopic approach, under one lung ventilation, using a double-lumen trachea-tube. Thoracoscopic surgery was performed in the right lateral position. The first trocar for the thoracoscope was placed at the seventh intercostal space over the midaxillary line. We then checked the thoracic cavity and the herniated organs. The left colon and spleen were located in the left thoracic cavity, as seen on the preoperative chest CT (Figure 3A and 3B). No hernia sac was found. We examined the herniated organs carefully. There was neither adhesion nor necrotic change. Second and third trocars were placed at the eighth intercostal space over the anterior and posterior axillary lines, respectively.

We used an Excel trocar^® ^for CO_2 _gas insufflation to increase intrathoracic pressure. The herniated organs, the left colon and spleen, were carefully returned to the abdominal cavity. These innovations, aimed at safely returning the herniated organs to the abdominal cavity, were performed with the patient in the head-up (reverse Trendelenburg) position with artificial pneumothorax. First, the patient was placed in the right lateral position and then he was shifted into a reverse Trendelenburg position. Whilst he was in this position, the artificial pneumothorax with CO_2 _gas was maintained at 8 cm H_2_O. The patient's circulatory and respiratory status was carefully monitored. These innovations facilitated safe hernia reduction. Fortunately, there were no adhesions in the left thoracic cavity. We were able to insert the thoracoscope through the diaphragmatic defect into the abdominal cavity and confirm the safe placement of the herniated organs.

There was neither torsion of the bowel nor bleeding in the abdominal cavity. The diaphragmatic defect, 5 cm × 6 cm in size, with a smooth circular edge was located posterolaterally (Figure 3C). The defect appeared to have gradually expanded at the thoracic wall. As he was a young man, we decided to perform primary closure of the diaphragmatic defect. We thought closing the defect of the diaphragm near the thoracic wall required unrolling and resuturing and we thought that it would be difficult to close the defect by thoracoscopic surgery alone. We thus added a small incision thoracotomy (5 cm in length) near the defect and repaired the diaphragm with a primary suture. The defect was closed using a single layer primary closure method with interrupted non-absorbable sutures (Figure 3D). The diaphragm near the thoracic wall required unrolling of the posterior diaphragmatic rim. After detachment, the defect near the thoracic wall was closed and again sutured to the thoracic wall. The thoracic cavity was drained with a single chest tube. The operative time was 144 min and there was no significant blood loss.

**Figure 4:**
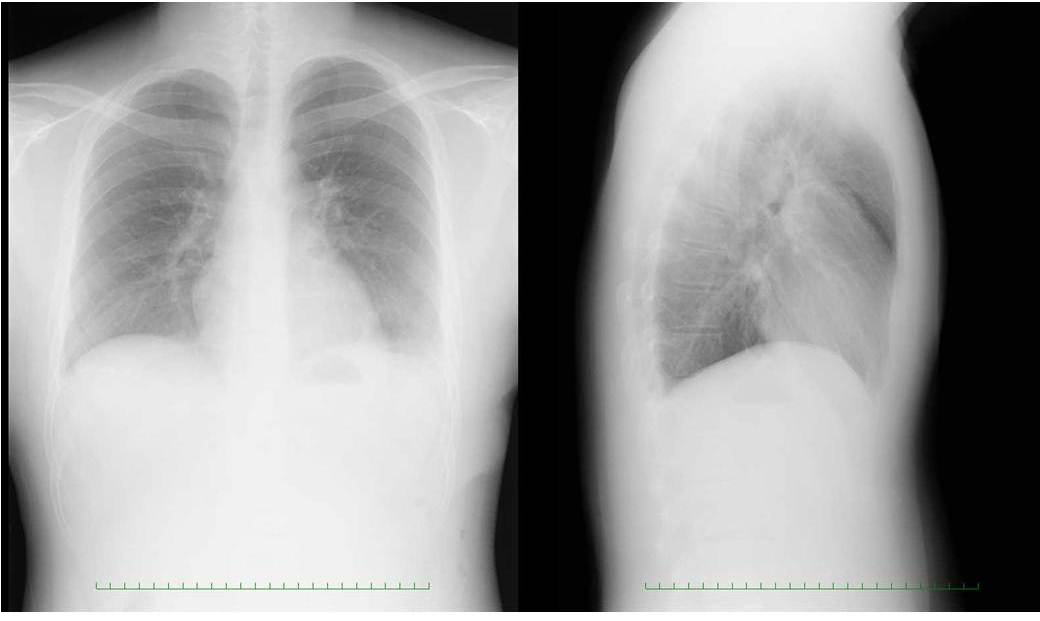
**Postoperative chest radiography**. There were no abnormalities on postoperative chest radiography.

**Figure 5:**
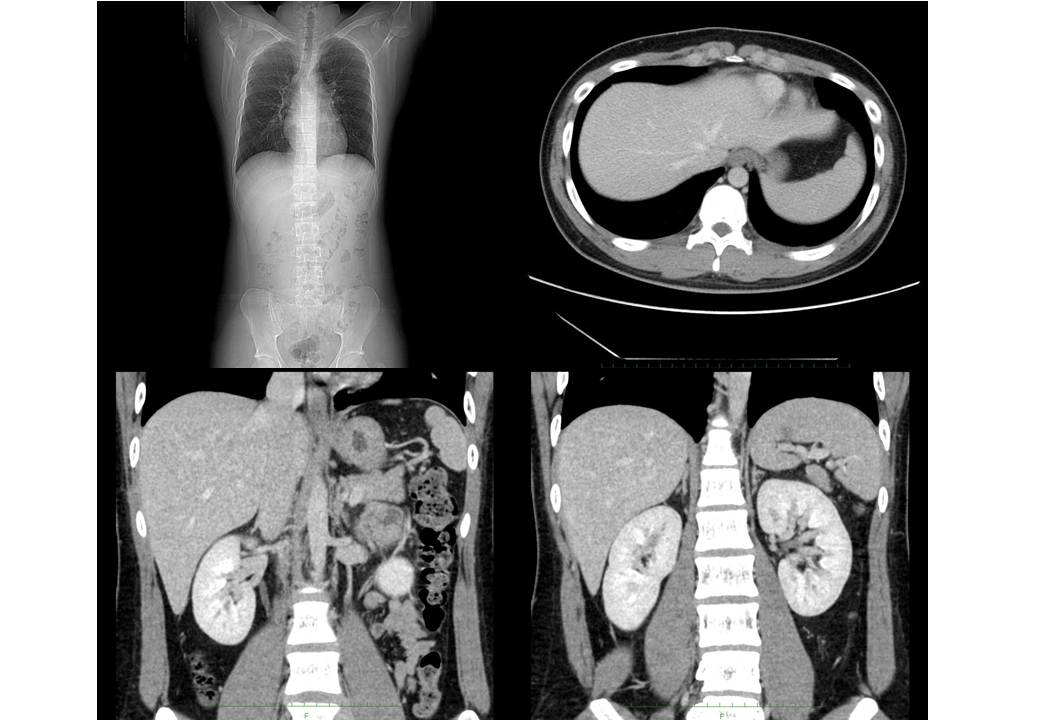
**Postoperative enhanced chest and abdominal computed tomography (CT) scans**. Two months postoperatively, an outpatient chest CT was performed. There was no recurrence of diaphragmatic hernia

The patient recovered uneventfully from anesthesia. A chest radiograph obtained 24 hours after surgery indicated adequate expansion of the left lung. On the first postoperative day (POD1), the chest tube was removed and he was put on a normal diet. After obtaining a final chest radiograph (Figure 4), he was discharged on POD5. Two months later an outpatient chest CT was performed and revealed that there had been no diaphragmatic hernia recurrence (Figure 5).

## Discussion

The incidence of CDH is reportedly 1 in 2200 to 12,500 live births and they occur more often on the left [7]. CDH was first described in 1679 by Lazarus Riverius, who incidentally noted a CDH during postmortem examination of a 24-year-old [8]. Bochdalek hernia is one of the CDHs first reported by Victor Alexander Bochdalek in 1848 [2]. The Bochdalek hernia has a female predominance and symptoms usually manifest during the first week of life [9]. Most Bochdalek hernias cause severe cardiorespiratory distress immediately after birth. Once diagnosed it is crucial to perform prompt surgical treatment.

The hernia is very rare in adults. The prevalence of asymptomatic cases in a large adult population, retrospectively reviewed with thin-slice CT scans, was only 0.17% based on 13,138 CT reports [4]. Among the surgical findings, a hernia sac was identified in 20% of patients [10,11]. All abdominal organs, except the rectum and genitals, have been found to have entered the thorax through a defect in the diaphragm: the colon, stomach, small bowel, omentum, spleen, kidney and even the tail of the pancreas [7,10,12-15].

The Bochdalek hernia is secondary to the incomplete development of the pleuroperitoneal folds due to improper or absent diaphragmatic muscle migration. The canals resulting from these folds are normally closed by pleuroperitoneal membranes in the eighth week of gestation. There are many symptoms of Bochdalek hernia. Typically, the diagnosis is based on dyspnea, recurrent chest infections and the absence of breath sounds in the thoracic region. In adults, gastrointestinal symptoms related to the obstruction of the herniated organ(s) are more common. These symptoms include abdominal pain, intestinal obstruction and chest tightness [4]. Herniated organs determine the symptoms. There are also reports of sepsis secondary to necrosis and perforation of a herniated colon [10,16].

Asymptomatic cases are difficult to diagnose. Bochdalek hernias in adults are usually detected incidentally during routine chest radiography. Frontal and lateral chest radiographs are the most important diagnostic tools [16]. Many Bochdalek hernias are identified by gas-filled bowel loops or a soft tissue mass above the dome of the diaphragm. However, if the herniation is intermittent, radiographs may appear normal. In addition, left middle lobe collapse, pneumonic consolidation, pericardial fat pad, pericardial cyst, mediastinal lipoma or an anterior mediastinal mass must be ruled out. A chest CT is necessary in order to make an accurate diagnosis. Chest CT shows the focal defect in the diaphragm, herniated contents and thickening of the diaphragm, or crus, as a result of edema or hematoma. Helical CT depicts these features even more clearly.

The conventional method is to return the herniated organs to the abdominal cavity and close the diaphragmatic defect through the thorax or the abdomen [5,17]. Thoracoscopic surgery facilitates the reduction of the herniated contents, allowing adhesion lysis and care of the herniated organs. With this procedure, bleeding control and diaphragmatic defect closure are easier and safer [5,6]. In addition to this procedure, the reverse Trendelenburg position and artificial pneumothorax facilitate the safe return of the herniated organs to their correct locations. Inflation-assisted bowel reduction with very low pressure for infants has been reported [18,19]. In our case, the artificial pneumothorax was maintained at 8 cm H_2_O under careful circulatory and respiratory monitoring. There was no change in cardiorespiratory status. With these innovations, the herniated organs were returned to the abdominal cavity. A treatment combination with laparoscopy, for examining the abdominal cavity, is very useful and reduces surgical morbidity. In our case, we were able to insert the thoracoscope through the diaphragmatic defect into the abdominal cavity. We confirmed the absence of ischemic change in the herniated organs and then closed the diaphragmatic defect with a primary suture. The patient was discharged on POD5 with minimal discomfort.

This procedure is useful not only for congenital diaphragmatic hernia but also for traumatic hernia, both blunt and penetrating. Generally, when the defect of the diaphragm is fairly large, tension-free repair using a prosthetic patch, such as composite or porcine mesh, is a very useful method which avoids a thoracotomy. We considered repairing it with a composite or porcine mesh but decided in this case to do a primary closure by suturing. The reasons for this were that: (1) our patient was still young; (2) repairing the diaphragmatic defect near the thoracic wall required unrolling and resuturing; and (3) there was no tension of the diaphragm. After unrolling of the posterior diaphragmatic rim, the defect of diaphragm was closed and again sutured to the thoracic wall under small thoracotomy without a prosthetic patch.

One of the advantages of a thoracoscopic repair of a Bochdalek hernia is that it is minimally invasive and the patient experiences less pain. In addition, the thoracic cavity and herniated organs can be examined in detail for ischemic change, necrosis and perforation. The presence of lung hypoplasia can also be confirmed. Thirdly, if herniated organs are attached to the thoracic wall or lung, lysis of the adhesions can be carried out safely.

However, there are disadvantages to the thoracoscopic procedure. First, it can be difficult to manipulate herniated organs. The spleen is especially prone to bleeding which is why we employed the reverse Trendelenburg position and artificial pneumothorax with CO_2 _gas insufflation. These innovations facilitated the safe return of the herniated organs to the abdominal cavity. Secondly, abdominal cavity visualization might be insufficient. We inserted the thoracoscope through the diaphragmatic defect into the abdominal cavity and were able to confirm the safe placement of the herniated organs.

## Conclusion

Bochdalek hernias are very rare in adults. We performed the surgical treatment under thoracoscopy. The reverse Trendelenburg position and artificial pneumothorax are useful innovations for reducing the herniated contents. The diaphragmatic defect was rather large. Generally, hernia repair using mesh is useful if one needs to avoid performing a thoracotomy. We considered this method but the patient was young; closing the diaphragmatic defect near the thoracic wall required unrolling and resuturing and there was no tension of the diaphragm which is necessary when resuturing. A small incision thoracotomy was therefore added and the primary closure of the diaphragmatic defect was performed. We inserted the thoracoscope through the diaphragmatic defect into the abdominal cavity and confirmed the safe placement of the herniated organs. Our patient was discharged on POD5. There has been no recurrence to date. We consider Bochdalek hernia repair with thoracoscopic-assisted surgery to be a safe and useful technique.

## Abbreviations

CDH: congenital diaphragmatic hernia; CT: computed tomography; POD: postoperative day.

## Competing interests

The authors declare that they have no competing interests.

## Consent

Written informed consent was obtained from the patient and the parent of the patient for publication of this case report and any accompanying images. A copy of the written consent is available for review by the Editor-in-Chief of this journal.

## Authors' contributions

NT, KT, TS and YM were the surgeons and attending physicians. HY and HO supplemented the data about case reports and analyzed the patient's data. NT and KT were the main contributors to the writing of the manuscript. All authors read and approved the final manuscript.
